# Costs of Surveillance Care After Definitive Treatment of Head and Neck Cancer: A Surveillance, Epidemiology, and End Results (SEER)-Medicare Analysis

**DOI:** 10.7759/cureus.114228

**Published:** 2026-08-09

**Authors:** Allison Verbyla, Sharvil C Desai, Gary V Walker, Matthew C Ward

**Affiliations:** 1 Biostatistics and Data Sciences, Levine Cancer Institute, Charlotte, USA; 2 Radiation Oncology, Banner MD Anderson Cancer Center, Gilbert, USA; 3 Radiation Oncology, Levine Cancer Institute, Charlotte, USA

**Keywords:** cancer surveillance, cancer survivorship, cost of care, head and neck squamous cell cancer, oropharynx cancer

## Abstract

Introduction: Following curative treatment of squamous carcinoma of the head and neck, surveillance care is indicated to detect recurrence. Historically, physical exams are the backbone of surveillance, but serial imaging and flexible endoscopy are also utilized. Recently, assays have become commercially available, which could aid in early detection of recurrence. To understand the potential economic impact of these assays, a baseline of surveillance costs must be established. We performed a Surveillance, Epidemiology, and End Results (SEER)-Medicare analysis to quantify the costs of classic surveillance procedures.

Materials and methods: We obtained data from the 2010-2017 SEER program linked to traditional Medicare Part A & B claims and enrollment information dating 2010-2019. Patients with non-metastatic cancers of the head and neck, including oral cavity, paranasal sinus, pharynx, and larynx, were identified. Definitive treatment with either surgery or radiation therapy (with or without systemic therapy) was required for inclusion. Patients were excluded if they had any lapse in insurance coverage (i.e., Parts A & B) or were enrolled in Medicare Advantage. The surveillance period was defined from 90 days past treatment through the date of death, hospice enrollment, or additional cancer treatments. Procedures related to routine surveillance were identified, including flexible endoscopy and imaging. The cumulative Medicare spend was quantified, adjusting for inflation to 2021 U.S. dollars.

Results: In total, 13,339 patients were identified; all were age ≥ 65. The most common site was the oropharynx (OPC), followed by larynx cancers. Of the OPC patients, 33% were human papillomavirus positive (HPV+), and for 55%, the HPV status was unknown. The median clinical follow-up was 43.0 months, and the median surveillance period was 16.6 months. The median costs were $1,246 (IQR $420-$2,381), with the top 5% incurring a cost of $7,657 (IQR $6,656-9,062). Surveillance was more expensive among younger, HPV+ OPC patients and for those who followed longer. Flexible laryngoscopy was the most frequent charge.

Conclusions: In an older population, payer costs follow a wide and skewed distribution but are highest among younger HPV+ OPC patients. These findings facilitate cost-effectiveness assessment of novel assays. Opportunity exists to reduce imaging and endoscopy spend by substituting more accurate biomarkers.

## Introduction

Head and neck cancer (HNC) presents a significant global health concern, accounting for over 790,000 new cases annually [1]. Despite therapeutic advances, recurrence rates remain high: up to 40% of patients experience disease recurrence after definitive treatment [2, 3]. Recurrence risk is highest in the initial post-treatment period and is influenced by factors such as tumor stage, human papillomavirus (HPV) status, and primary site [4].

Current surveillance strategies for HNC survivors are guided by consensus recommendations from organizations such as the National Comprehensive Cancer Network (NCCN), American Cancer Society (ACS), and the American College of Radiology (ACR), which advocate for regular history and physical examination, with imaging reserved for symptomatic patients or those with abnormal findings [5-8]. The recommended frequency of clinical follow-up is every one to three months in the first year, every two to six months in the second year, and less frequently thereafter. However, the utility of routine imaging in asymptomatic patients is increasingly questioned, as multiple studies have failed to demonstrate a survival benefit or improved salvage rates with scheduled imaging compared to clinical examination alone [2, 9-11]. Routine surveillance imaging may contribute to unnecessary financial burden, radiation exposure, and resource utilization without clear evidence of benefit. Therefore, recent guidelines suggest that surveillance should be individualized based on recurrence risk, with only higher-risk patients for whom the tumor site is difficult to visualize considered for serial imaging [5, 12].

Moving beyond serial imaging, emerging evidence now supports the use of circulating tumor DNA (ctDNA) and tumor tissue-modified viral (TTMV)-DNA assays as highly sensitive and specific tools for the early detection of recurrence, particularly in HPV-driven oropharyngeal squamous cell carcinoma (OPSCC) [13-15]. These assays can be performed serially and have demonstrated high positive and negative predictive values, often identifying recurrence months before clinical or radiologic detection [16].

While promising, concerns over the costs of serial measurement have slowed the adoption of these biomarkers [17]. Initial cost-effectiveness modeling suggests that strict adherence to biomarkers instead of imaging may be economically feasible [17, 18]. However, the validity of any cost-effectiveness model is contingent upon the baseline costs of the reference (traditional surveillance modalities), which have not been well quantified. Herein, we used the Surveillance, Epidemiology, and End Results (SEER)-Medicare database to quantify the costs of surveillance care in the pre-biomarker era, providing a foundation for a value assessment of novel assays used in survivorship.

This work was presented in digital poster format at the 2025 American Society of Radiation Oncology (ASTRO) meeting.

## Materials and methods

Data source

Formed in 1973, the SEER program is a set of population-based registries that was expanded from 18 to 21 geographical areas in 2018 and covers approximately 35% of the United States population [19]. Through a de-identified process, approximately 96% of cancer cases held in the SEER database are linked to Medicare claims data, forming a relational database queryable for cost of care. For this study, files obtained included (1) SEER Cancer Data, Medicare Enrollment Master Beneficiary Summary File (MBSF); (3) Medicare Provider Analysis and Review (MEDPAR) file; (4) National Claims History (NCH) file; (5) Outpatient file; and (6) Hospice file. Atrium Health Institutional Review Board approval was waived, and a Data Use Agreement was executed. This report conforms to STrengthening the Reporting of OBservational studies in Epidemiology (STROBE) guidelines for observational research [20].

Study cohort

We obtained data from the 2010-2017 SEER program linked to traditional Medicare Part A & B claims and enrollment information dating from 2010-2019. Patients age 65 and older, with a diagnosis date of cancer from 2010 to 2017 with non-metastatic, pathologically confirmed cases of squamous cell carcinoma originating from mucosal subsites of the head and neck, including oral cavity, paranasal sinus, pharynx, and larynx, were identified. Patients were excluded if they had any lapse in insurance coverage (i.e., Parts A & B) or were enrolled in Medicare Advantage (Part C). 

Outcomes

Healthcare Common Procedure Coding System (HCPCS) codes were utilized to differentiate the phases of care, defined as treatment, surveillance, and recurrence [21]. The “surveillance” period was defined from 90 days past treatment through the date of death, hospice enrollment, or additional cancer treatments. Treatment was defined using HCPCS codes related to surgical, radiation, or chemotherapy treatments (Table 1).

**Table 1:** Healthcare Common Procedure Coding System (HCPCS) Codes Defining the Treatment Phase Reference: [21]

Category	Procedure / Drug	HCPCS Code(s)
Surgery		
Surgery	Surgical procedures on the pharynx, adenoids, and tonsils	42700-42999
Surgery	Surgical procedures on the larynx	31300-31599
Surgery	Surgical procedures on the tongue and floor of mouth	41000-41599
Surgery	Surgical procedures on the lymph nodes and lymphatic channels	38300-38780 (excluding non–head and neck sites: 38740, 38745, 38746, 38747, 38760, 38765, 38770, 38780)
Radiation		
Radiation	Radiation oncology treatment	77261-77799
Chemotherapy administration		
Chemotherapy	Hydration, therapeutic/prophylactic/diagnostic injections and infusions, and chemotherapy and other highly complex drug or biologic agent administration	96360-96549
Chemotherapy drugs (J codes)		
Drug	Carboplatin injection	J9045
Drug	Cetuximab injection	J9055
Drug	Cisplatin 10 mg injection	J9060
Drug	Docetaxel injection	J9171
Drug	Fluorouracil injection	J9190
Drug	Methotrexate sodium injection	J9250
Drug	Methotrexate sodium injection	J9260
Drug	Paclitaxel injection	J9265
Drug	Paclitaxel injection	J9267
Drug	Pembrolizumab injection	J9271
Drug	Nivolumab injection	J9299
Drug	Atezolizumab injection, 10 mg	J9022
Drug	Avelumab injection, 10 mg	J9023

Note that oral chemotherapies (filed under Medicare Part D) were not included due to a limited role in the management of head and neck cancer. 

Costs

The primary outcome was the cumulative costs of surveillance-related procedures. Procedures related to routine surveillance were identified, including flexible endoscopy and imaging. Imaging procedures included in the cost analysis are listed in Table 2. 

**Table 2:** Healthcare Common Procedure Coding System (HCPCS) Codes Defining the Surveillance Phase CT: computed tomography; MRI: magnetic resonance imaging; PET: positron emission tomography; W: with; WO: without. Reference: [21]

HCPCS Code	Description
70460	CT head W contrast
70450	CT head WO contrast
70470	CT head WWO contrast
70487	CT maxillofacial W contrast
70486	CT maxillofacial WO contrast
70488	CT maxillofacial WWO contrast
70491	CT neck W contrast
70490	CT neck WO contrast
70492	CT neck WWO contrast
70543	MRI orbit, face, neck WWO contrast
70542	MRI orbit, face and/or neck W contrast
70540	MRI orbit, face, neck WO contrast
78815	PET/CT W or WO contrast
71250	CT chest WO contrast
71260	CT chest W contrast
71270	CT chest WWO contrast
74176	CT abdomen/pelvis WO contrast
74177	CT abdomen/pelvis W contrast
74178	CT abdomen/pelvis WWO contrast
31575	Laryngoscopy, flexible, diagnostic

The cumulative Medicare payment from these services was quantified by summing all paid claims related to surveillance HCPCS codes with a date of service contained within the defined surveillance period (the period from 90 days post treatment until the next treatment, hospice enrollment, or death). 

For hospital services, this amount does not include the claim pass-through per diem payments made by Medicare. To obtain the total amount paid by Medicare for the claim, the pass-through amount (which is the daily per diem amount) must be multiplied by the number of Medicare-covered days and added to the claim payment amount (this field), a correction that we performed.

For non-hospital (and hospital outpatient) services and for other non-institutional services (carrier and Durable Medical Equipment (DME)), this variable equals the total actual Medicare payment amount, and pass-through amounts do not apply.

For Part B non-institutional services (carrier and DME), this variable equals the sum of all the line item-level Medicare payments as listed in the National Claims History file.

Claims were adjusted for inflation to 2021 U.S. dollars using the Consumer Price Index (CPI) [22].

Statistical analysis

SAS input programs (SAS Institute Inc., Cary, NC, USA) provided by the SEER program were used to import six provided files, which included: (1) SEER Cancer Data, (2) Medicare Enrollment MBSF file, (3) MEDPAR, (4) NCH, (5) Outpatient, and (6) Hospice files. All files were combined by setting type and patient ID. Table 3 presents the full list of studied variable names.

**Table 3:** Analysis Variables Utilized SEER: Surveillance, Epidemiology, and End Results; HPV: human papillomavirus; NA: not applicable; AJCC: American Joint Committee on Cancer; ICD = International Classification of Diseases; CS: Collaborative Stage; COD: cause of death; DRG: diagnosis-related group; SNF: skilled nursing facility; TNM: tumor, node, metastasis

Variable	Description
Patient_ID	Patient ID
SEER_Registry	Registry origin
Marital_status_at_diagnosis	Marital status
Race_ethnicity	Race/ethnicity
Sex	Sex assigned by registry
Age recode with single ages_and_100	Age at diagnosis
Sequence_number	Cancer sequence number
Month_of_diagnosis	Month of diagnosis
Year_of_diagnosis	Year of diagnosis
Month_of_diagnosis_recode	Recode of month of diagnosis
Primary_Site	Primary site of index cancer
Laterality	Laterality of cancer
Histologic_Type_ICD_O_3	Histology type
Behavior_code_ICD_O_3	Behavior code (benign/malignant)
Grade	Grade of the cancer
Diagnostic confirmation	Method of diagnosis
Type of reporting source	Reporting source
Regional nodes positive	Regional nodes positive
Regional nodes examined	Regional nodes examined
CS Mets at Dx	Metastasis at diagnosis
CS Lymph nodes	Lymph nodes at diagnosis
CS Site-specific factor 10	Site-specific factor 10 (HPV status)
CS Version input current	Collaborative stage version
RX sum surg prim site	Surgery to primary site
RX sum surg/rad se	Sequence of surgery and radiation
RX sum reg ln examined	Summary count of regional nodes examined
Reason no cancer directed surgery	Rationale for omission of surgery
Radiation Recode	Radiation use
Chemotherapy recode	First-course chemotherapy
Site specific surgery	Legacy site-specific surgery field (1998-2002)
Scope of reg lymph and surg	Extent of regional lymph node surgery
Site recode ICD-O-3	SEER site grouping derived from ICD-O-3 topography/histology
First malignant primary indicator	Indicator for whether the tumor is the patient's first malignant primary
COD to site recode	Cause of death recoded to a SEER cancer-site grouping
COD to site rec km	Cause-of-death recode constructed for Kaplan-Meier cause-specific survival
Vital status recode	Patient's vital status at end of follow-up (alive vs dead)
Summary stage 2000	Summary Stage 2000
Survival months	Months from date of diagnosis to date of death or last follow-up
Survival months flag	Quality flag for the survival-months calculation
Derived AJCC T & N & M 7th Ed	T, N, and M categories derived by SEER from CS inputs using AJCC 7th edition rules
Derived AJCC Stage Group	AJCC stage group derived from the T/N/M above
SEER combined mets at Dx Bone	Presence of bone metastases at diagnosis (yes/no/unknown)
Brain	Presence of brain metastases at diagnosis (2010+)
Liver	Presence of liver metastases at diagnosis (2010+)
Lung	Presence of pulmonary metastases at diagnosis (2010+)
Derived Combined TNM 2016+	T/N/M and stage group under the post-2016 AJCC system
CS Site Specific Factors #1-6	For the H&N schemas: SSF1 = size of involved lymph nodes; SSF2 = extracapsular extension, clinical; SSF3 = extracapsular extension, pathologic; SSF4-6 = lymph node levels/groups involved (location relative to cricoid, levels I-VII, retropharyngeal)
Rx Summ Radiation	Radiation use during first course
SEER Date of Death	Month and year of death (day is suppressed in research files)
SEER Date of Follow-up	Date of last contact / last known alive (used as censoring date for living patients)
Year of Birth	Year of birth
Date of Diagnosis Flag	Flag describing completeness/imputation of the diagnosis date
Date of Therapy Started Flag	Flag describing completeness/imputation of the therapy-start date
Date of Birth Flag	Flag describing completeness/imputation of the birth date
Date therapy started	Date first-course treatment was initiated
Rx Summ Other	First-course 'other' therapy: captures non-standard modalities not falling under surgery/radiation/chemotherapy
HPV Recode (2010+)	HPV status field that recodes CS Site-Specific Factor 10 into three categories: HPV Positive, HPV Negative, Unknown/NA
Yost 2010-2017 quintile	Census tract-level composite socioeconomic status quintile
Medicare claims variables (MEDPAR / claims files)	
BENE_MLG_CNTCT_ZIP_CD	Zip code of the beneficiary’s residence
SRC_IP_ADMSN_CD	Source of admission to an inpatient facility - for newborn admit is type of delivery code
IP_ADMSN_TYPE_CD	Inpatient admission type code
ADMSN_DT	Date beneficiary admitted for inpatient care or date care started
DSCHRG_DT	Date beneficiary was discharged or died
LOS_DAY_CNT	Days of beneficiary’s stay in a hospital/SNF
UTLZTN_DAY_CNT	Covered days of care chargeable to Medicare utilization for stay
BENE_PTA_COINSRNC_AMT	Beneficiary’s liability for Part A coinsurance for stay ($)
BENE_IP_DDCTBL_AMT	Beneficiary’s liability for deductible for stay ($)
BENE_BLOOD_DDCTBL_AMT	Beneficiary’s liability for blood deductible for stay ($)
DRG_OUTLIER_STAY_CD	DRG cost or day outlier code
DRG_OUTLIER_PMT_AMT	DRG outlier approved payment amount ($)
DRG_PRICE_AMT	DRG price amount ($)
ACMDTNS_TOT_CHRG_AMT	Total charge for all accommodations ($)
RDLGY_CHRG_AMT	Radiology charge amount (excluding MRI) ($)
ER_CHRG_AMT	Emergency room (ER) charge amount ($)
ICU_IND_CD	Intensive care unit (ICU) indicator code
CRNRY_CARE_IND_CD	Coronary care unit (CCU) indicator code
RDLGY_ONCLGY_IND_SW	Oncology indicator
RDLGY_THRPTC_IND_SW	Therapeutic radiology indicator
RDLGY_NUCLR_MDCN_IND_SW	Radiology nuclear medicine indicator
RDLGY_CT_SCAN_IND_SW	Radiology computed tomographic (CT) scan indicator
ADMTG_DGNS_CD	Initial diagnosis at time of admission
ADMTG_DGNS_VRSN_CD	Admitting diagnosis version code (ICD-9 or ICD-10)
DGNS_1_CD	Principal diagnosis code
SRGCL_PRCDR_IND_SW	Surgical procedure indicator
SRGCL_PRCDR_CD_CNT	Surgical procedure codes included in stay
SRGCL_PRCDR_VRSN_CD	Surgical procedure version code (earlier version)

We selected claims to be used by starting with only therapy and surveillance claims; no other types of claims were included. We selected only patients in the cohort who had a 90-day window free of treatment, indicating the patient had started a surveillance period for subsequent head and neck cancers. Patients without a surveillance period were counted to have a payment of zero but left in the analysis to ensure an accurate denominator. 

Analysis of cost patterns examined payments by age, race, ethnicity, diagnosis year, cancer site, and HPV status. Additionally, analyses were performed to assess Medicare payments by HPV status for oropharyngeal cancers alone as well as by secondary cancer treatment (surgery and radiation), and year of surveillance care. Lastly, average frequency of payments for each HCPCS code (n>15) in the surveillance period was analyzed across all patients in the study population. 

Descriptive statistics are used primarily for the purpose of calculating cumulative payments. No advanced regressions or analyses were utilized. All analyses were performed in SAS 9.4 and R (R Foundation for Statistical Computing, Vienna, Austria).

## Results

**Figure 1:**
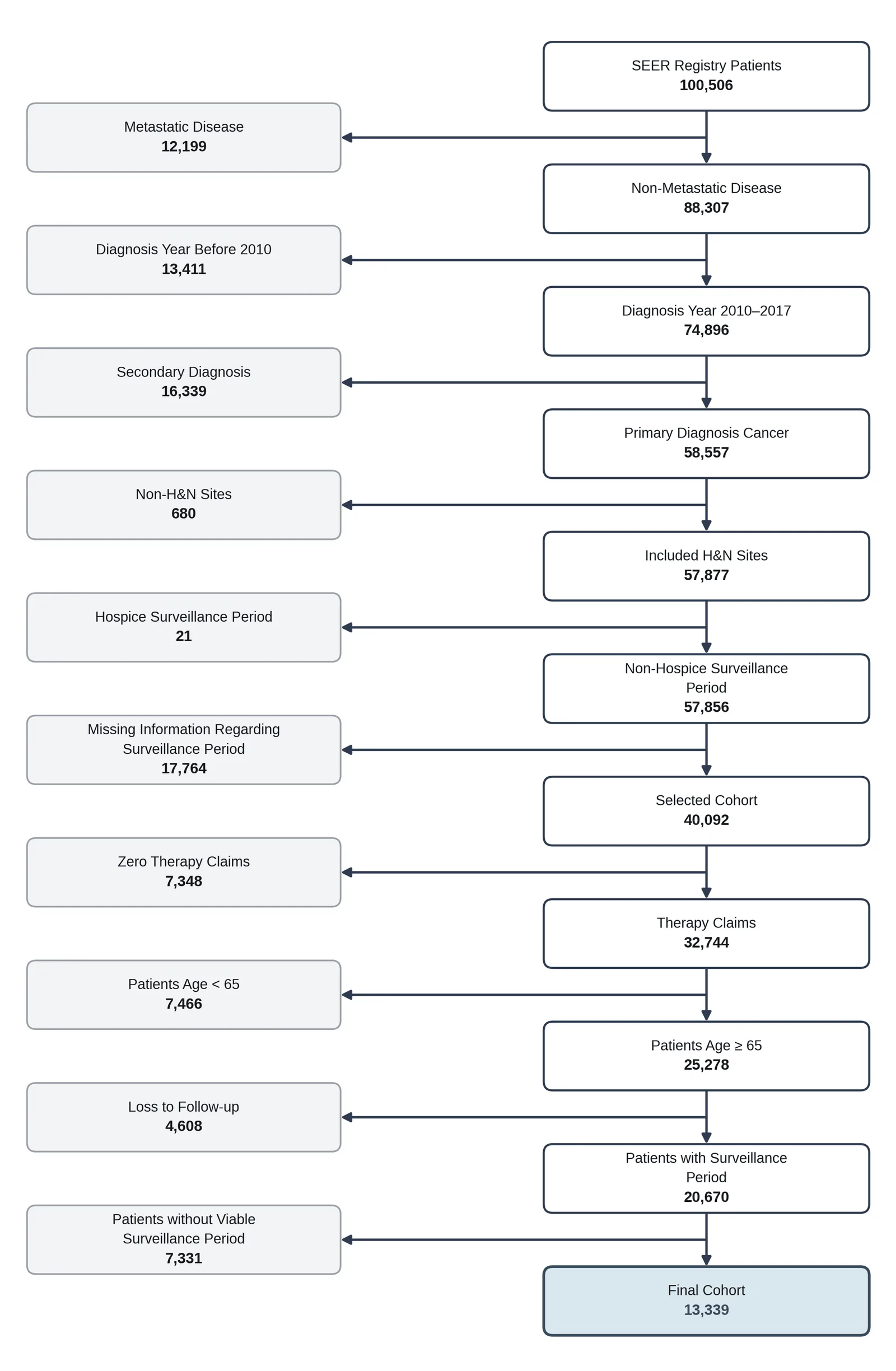
STROBE Diagram STROBE: STrengthening the Reporting of OBservational studies in Epidemiology; SEER: Surveillance, Epidemiology and End Results program; H&N: head and neck.

In total, 13,339 patients were identified; all were aged ≥65. The most common site was oropharynx (OPC), followed by larynx primaries. Of the OPC patients, 34% were known HPV positive (HPV+). Table 4 displays the characteristics of patients included (patient, disease & treatment factors), and Figure 1 demonstrates the STROBE diagram of the cohort formation.

**Table 4:** Cohort Demographics HPV: human papillomavirus Note: Race and ethnicity categories originated in the source SEER dataset.

Characteristic	Number (N)	Percentage (%)
Age (years)		
65-69	4,773	35.8
70-79	5,578	41.8
80+	2,988	22.4
Sex		
Male	9,402	70.5
Female	3,937	29.5
Race		
White	11,590	86.9
Black	943	7.1
Asian	537	4.0
Unknown	79	0.6
Other Asian	68	0.5
American Indian	52	0.4
Pacific Islander	38	0.3
Other	32	0.2
Ethnicity		
Non-Spanish-Hispanic-Latino	12,554	94.1
Spanish-Hispanic-Latino	785	5.9
Diagnosis Year		
2010	1,604	12.0
2011	1,636	12.3
2012	1,769	13.3
2013	1,811	13.6
2014	1,900	14.2
2015	1,839	13.8
2016	1,387	10.4
2017	1,393	10.4
HPV Status		
HPV negative	613	4.6
HPV positive	1,310	9.8
Unknown/NA	1,806	13.5
Not 2013+ or selected schema	6,603	49.5
Missing	3,007	22.5
Site Group		
Oropharynx	3,806	28.5
Larynx	3,762	28.2
Oral cavity	3,064	23.0
Major salivary	1,279	9.6
Sinonasal	610	4.6
Hypopharynx	477	3.6
Nasopharynx	341	2.6

Surveillance period

The median clinical follow-up was 43.0 months (IQR 21-69), and the median surveillance period was 16.6 months (IQR 6-36). The most common reasons for discontinuing surveillance were additional treatment or hospice enrollment.

Cumulative costs

The median costs for the entire cohort were $1,246, and the arithmetic mean was $1,841, with an IQR of $420-$2,381 (Table 5). These costs varied widely and demonstrated a rightward skew, with the top 5% of patients incurring significantly higher costs (median $7,657, IQR $6,656-9,062). 

**Table 5:** Surveillance Costs by Subset HPV: human papillomavirus; OPC: oropharynx cancer Note: Race and ethnicity categories originated in the source SEER dataset.

Characteristic	N	Medicare Payment/Patient (Median)	IQR
All patients	13,339	$1,246	$412-$2,381
Age (years)			
65-69	4,773	$1,485	$515-$2,895
70-79	5,578	$1,296	$421-$2,459
≥80	2,988	$857	$329-$1,785
Sex			
Male	9,402	$1,323	$437-$2,547
Female	3,937	$1,082	$383-$2,164
Race			
White	11,590	$1,280	$426-$2,455
Black	943	$1,077	$312-$2,051
Asian	537	$1,577	$548-$2,803
Other Asian	68	$1,351	$458-$2,475
American Indian	52	$1,153	$271-$1,765
Pacific Islander	38	$1,047	$435-$2,473
Unknown	79	$822	$347-$2,589
Other	32	$741	$239-$1,393
Ethnicity			
Non-Spanish-Hispanic-Latino	12,554	$1,272	$422-$2,432
Spanish-Hispanic-Latino	785	$1,172	$378-$2,318
Site Group			
Hypopharynx	477	$1,674	$687-$3,058
Larynx	3,762	$1,029	$352-$2,108
Major salivary	1,279	$1,050	$395-$2,058
Nasopharynx	341	$2,596	$550-$2,989
Oral cavity	3,064	$1,616	$319-$1,867
Oropharynx	3,806	$1,763	$730-$3,355
Sinonasal	610	$1,133	$384-$2,254
Medicare Payments per post-treatment year			
No Claims	2,488	$0	-
Costs in year 1 post treatment	10,851	$1,069	$344-$2,001
Costs in year 2 post treatment	5,071	$622	$277-$1,364
Costs in year 3 post treatment	3,074	$477	$218-$1,045
HPV Status			
HPV+ OPC	1,248	$1,924	$903-$3,702
HPV- OPC/Unknown	1,750	$1,540	$580-$2,882
Receipt of Surgery			
Surgery	4,207	$1,337	$469-$2,602
No surgery	9,132	$1,235	$401-$2,355
Receipt of Radiation			
Radiation	6,488	$1,878	$930-$3,556
No radiation	6,851	$703	$276-$1,620

Costs differed across demographic groups. Younger patients had higher expenditures, with a median of $1,485 among those aged 65-69 compared to $857 for those aged ≥80. Male patients had slightly higher costs than female patients ($1,323 vs. $1,082). Differences by race were modest, although Asian patients demonstrated somewhat higher median costs ($1,577), while Black patients incurred lower median costs ($1,077).

Nasopharyngeal ($2,596), oropharyngeal ($1,763), and hypopharyngeal ($1,674) cancers had the highest median costs, whereas laryngeal cancers had lower costs ($1,029), likely reflecting easier visualization on physical examination. HPV+ oropharyngeal cancer was associated with higher surveillance costs ($1,924, IQR $903-$3,702), consistent with longer survival and extended follow-up in this subgroup. Treatment modality also influenced costs; receipt of radiation therapy was associated with substantially higher expenditures compared to no radiation ($1,878 vs. $703), while surgery was associated with only modest differences ($1,337 vs. $1,235).

Temporal patterns demonstrated that surveillance spending was front-loaded. Median costs were highest in the first post-treatment year at $1,069 (IQR $344-$2,001), decreasing to $622 (IQR $277-$1,364) in year 2 and $477 (IQR $218-$1,045) in year 3. Across all groups, flexible laryngoscopy was the most frequently billed service, while imaging, though less frequent, contributed disproportionately to higher cumulative costs, particularly among high-cost outliers.

## Discussion

In this study, we establish a “real-world” baseline for surveillance costs in the era prior to novel biomarkers such as TTMV-ctDNA, using a population-based dataset representative of older adults. We found that a significant rightward skew in costs exists and that compliance with routine surveillance may be limited, with many patients incurring zero costs related to scans or exams. Overall, this is a representative sample that suggests surveillance care is likely less of a financial burden than treatment costs and that novel biomarkers do have the potential to increase Medicare costs as adoption increases.

Comparing our estimate to other assessments, our projection appears credible. One study by Mariotto et al. reported “continuing phase” care in 2019 U.S. dollars to be $5,200, including all charges [23]. Our paper limits charges, which may be related to care of head and neck cancer directly; thus, the average estimate seems comparable. In our previous cost-effectiveness analysis, we modeled the Medicare spend over five years to be an estimated $4,760 (2020 US dollars) based on the publicly available Medicare physician fee schedule. In this current report, most “real-world” costs were over the first three years, after which compliance dropped significantly and thus reduced the overall costs [17, 24].

The implications of this analysis are notable for economic evaluations of surveillance protocols. First, "real-world" Medicare spending on surveillance is likely dwarfed by treatment costs, particularly when considering novel pharmaceuticals and costs of hospitalization. Second, compliance with surveillance is likely limited (particularly in an older population), and patients may not receive all the “per protocol” assays beyond two to three years. Finally, these parameters can be used to improve the accuracy of additional cost-effectiveness assessments, which may guide more efficient health policies in the future.

There are notable limitations to this study. First, this is an older cohort aged 65 and above, and it is possible that real-world utilization and compliance may be more common in a younger population. Second, the rationale for each HCPCS charge is not available in the data, and some "overcounting" of scan charges is likely. Third, enrollment in Medicare Part C (Medicare Advantage) has increased steadily over the years, threatening the generalizability of the traditional Medicare Part B sample. Additionally, 18.6% of patients did not have any claims, which could represent follow-up not captured or incomplete data. A combination of these limitations of the SEER-Medicare program likely contributed to the short surveillance follow-up (median 16.6 months) despite the longer survival follow-up (median 43.0 months). In other words, the claims data did not fully match the survival data, a limitation of SEER-Medicare. Finally, the study included patients treated between 2010 and 2017. While this was performed to be representative of a population prior to the utilization of novel biomarkers, it is conceivable that practice patterns have changed such that utilization of scans may be higher today.

## Conclusions

In an older population, United States payer spending on surveillance care follows a wide and skewed distribution but is highest among younger HPV-associated OPC patients. Future cost-effectiveness assessments of novel biomarkers can utilize this information, obtained in the pre-ctDNA era, to inform parameter inputs and sensitivity analyses.

Opportunity exists to reduce imaging and endoscopy spend by substituting more accurate biomarkers such as ctDNA, though this may increase Medicare expenses overall. Unrestricted serial use of more costly biomarkers may not demonstrate cost-effectiveness compared to the historical standard, given the low costs observed in this study. However, if restricted to a higher-risk population, it may be possible to demonstrate the cost-effectiveness of serial biomarkers.
